# Rescue balloon pulmonary angioplasty for life-threatening acute pulmonary embolism on chronic thromboembolic pulmonary hypertension patients

**DOI:** 10.1016/j.rmcr.2021.101415

**Published:** 2021-04-13

**Authors:** Keiko Sumimoto, Yu Taniguchi, Hiroyuki Fujii, Keisuke Miwa, Yoichiro Matsuoka, Yasunori Tsuboi, Noriaki Emoto, Ken-ichi Hirata

**Affiliations:** Division of Cardiovascular Medicine, Department of Internal Medicine, Kobe University Graduate School of Medicine, Kobe, Japan

**Keywords:** Chronic thromboembolic pulmonary hypertension, Rescue balloon pulmonary angioplasty, Acute pulmonary embolism, Veno-arterial extracorporeal membrane oxygenation

## Abstract

We report the cases of two patients with life-threatening acute pulmonary embolism (PE) on chronic thromboembolic pulmonary hypertension (CTEPH) who were treated with rescue balloon pulmonary angioplasty (BPA). These cases highlight the effect of rescue BPA on acute PE on CTEPH, which requires veno-arterial extracorporeal membrane oxygenation.

## Highlights

• Chronic thromboembolic pulmonary hypertension (CTEPH) is a fatal disease, which may deteriorate rapidly and require mechanical support.
• Rescue ballon pulmonary angioplasty (BPA) may be effective for diatal-type CTEPH patients requiring mechanical support.
• Rescue BPA should be consdered as an option in the absence of treatment for critical CTEPH patients.

## 1 Introduction

Chronic thromboembolic pulmonary hypertension (CTEPH) is characterized by stenosis and obstruction of pulmonary arteries with non-resolving organized thromboemboli, leading to severe pulmonary hypertension (PH) and right heart failure [1]. It is categorized as Group 4 in the updated clinical classification of PH proposed in the recent 2018 world symposium [2]. The condition of CTEPH patients sometimes deteriorates with recurrent episodes of acute pulmonary embolism (PE) [3]. Pulmonary endarterectomy (PEA) remains the gold standard treatment for operable CTEPH. Balloon pulmonary angioplasty (BPA), an endovascular procedure to widen the narrowed or obstructed pulmonary arteries that are surgically inaccessible, would be a promising treatment option for patients with non-operable CTEPH. However, only limited reports have supported the efficacy and safety of rescue BPA in patients with very severe conditions requiring extracorporeal membrane oxygenation (ECMO) support. Here, we report the cases of successful rescue BPA for acute PE on CTEPH with ECMO.

## 2 Case 1

**Fig. 1:**
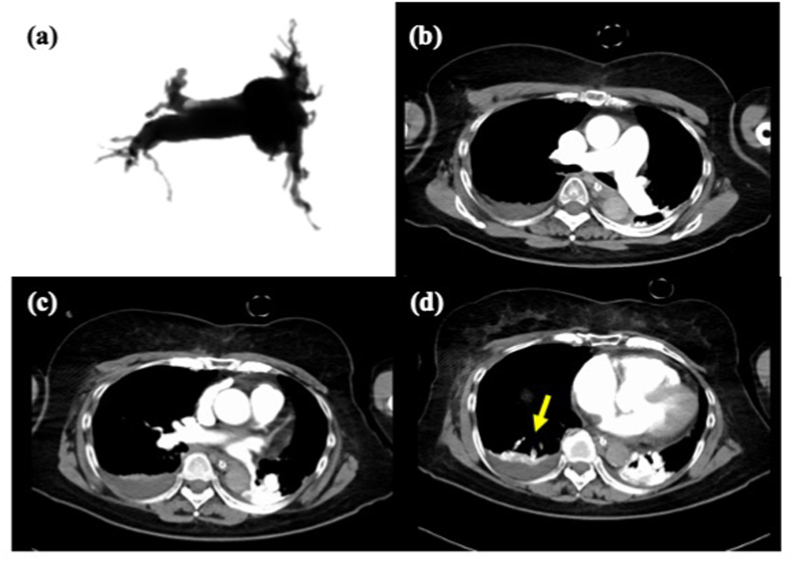
The computed tomography (CT) pulmonary angiography showed a small fresh thrombus in the right A10 (yellow arrow) and chronic thromboembolic obstruction of the bilateral distal pulmonary artery. (For interpretation of the references to colour in this figure legend, the reader is referred to the Web version of this article.)

**Fig. 2:**
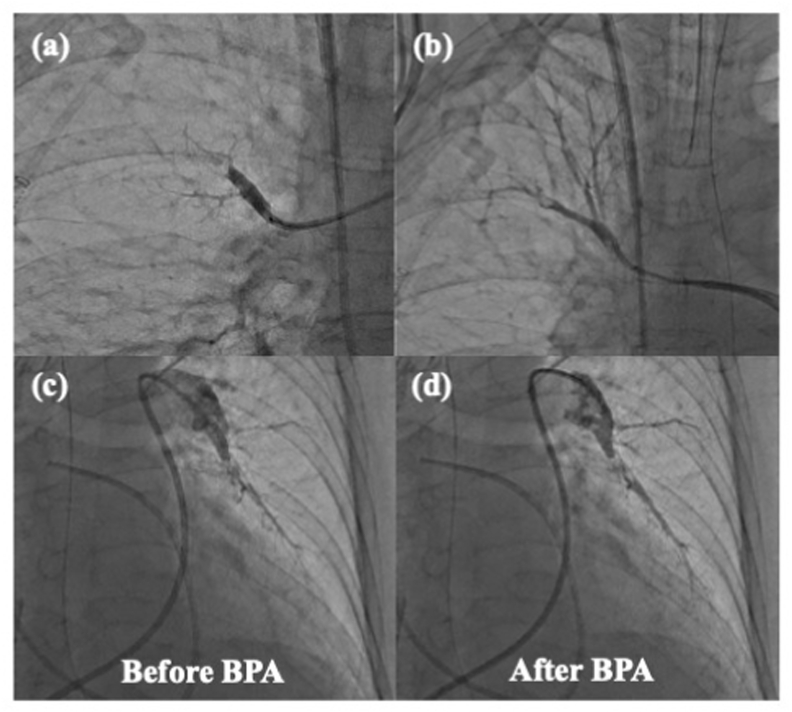
The comparison of pulmonary angiography. (a): pulmonary angiography of right pulmonary artery before BPA; (b): pulmonary angiography of right pulmonary artery after BPA.; (c): pulmonary angiography of left pulmonary artery before BPA; (d): pulmonary angiography of left pulmonary artery after BPA. We increased the pulmonary flow slightly not to cause high perfusion injury or balloon injury.

A 70-year-old woman was diagnosed with acute PE and treated with an anticoagulant 2 years ago. When she developed chronic subdural hematoma 2 weeks after the initiation of the anticoagulant, she discontinued taking the medication. She reported progressive exertional dyspnea in the past few months and was admitted to the hospital. Echocardiography revealed severe PH with a *trans*-tricuspid pressure gradient (TR-PG) of 91 mmHg, and laboratory investigations showed a D-dimer level of 3.2 μg/mL. Her condition rapidly deteriorated, and she presented with cardiorespiratory arrest. The patient's spontaneous circulation resumed soon after cardiopulmonary resuscitation. However, as her hemodynamic state was unstable, ECMO was instituted. Right heart catheterization (RHC) performed with ECMO support of 2.5 L/min revealed very severe PH, mean pulmonary artery pressure (PAP) 56 mmHg, cardiac output 1.03 L/min, and pulmonary vascular resistance (PVR) 48.5 wood units. Computed tomography (CT) pulmonary angiography showed a small fresh thrombus in the right A10 and chronic thromboembolic obstruction of the bilateral distal pulmonary artery (Fig. 1). Even with systemic heparinization for 3 days, no hemodynamic improvement was observed; mean PAP 54 mmHg. Therefore, we decided to perform rescue BPA. Although there was a small fresh bloody thrombus in the right pulmonary artery (A10), the rest was a distal organized thrombus. We performed BPA in the right (A1, A3, A5, A7, A8, A10) and left (A5, A8, A9, A10) pulmonary arteries with a small balloon with a diameter of 2.0 and 3.0 mm (Fig. 2). Soon after rescue BPA, her cardiorespiratory situation improved. ECMO was successfully discontinued the day after BPA, and she was extubated 3 days after BPA. The results of RHC performed 15 days after BPA were as follows: mean PAP 27 mmHg and PVR 5.3 wood units. Twenty-three days after BPA, she was discharged with anticoagulation therapy.

## 3 Case 2

**Fig. 3:**
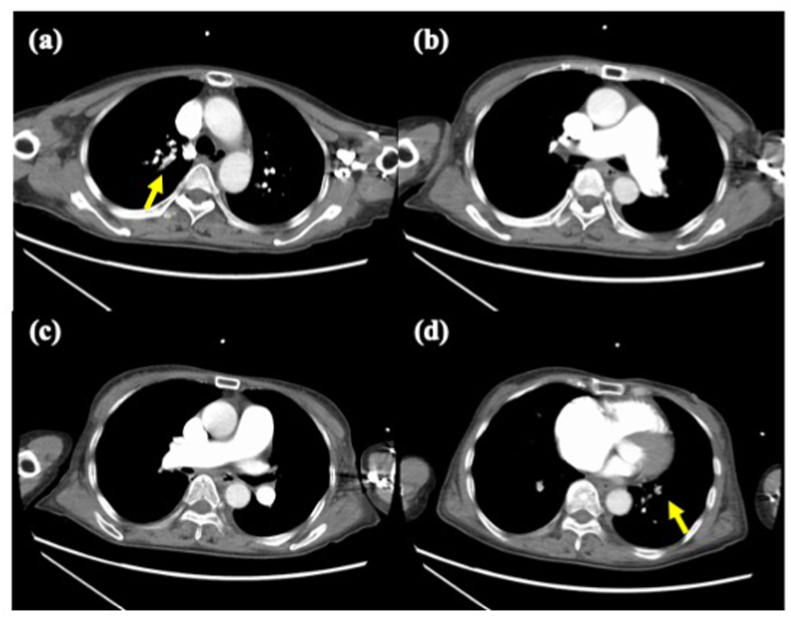
The enhanced CT showed a small defect of fresh thrombus (yellow arrows) in the bilateral pulmonary arteries at the segmental or subsegmental levels and signs of CTEPH. (For interpretation of the references to colour in this figure legend, the reader is referred to the Web version of this article.)

**Fig. 4:**
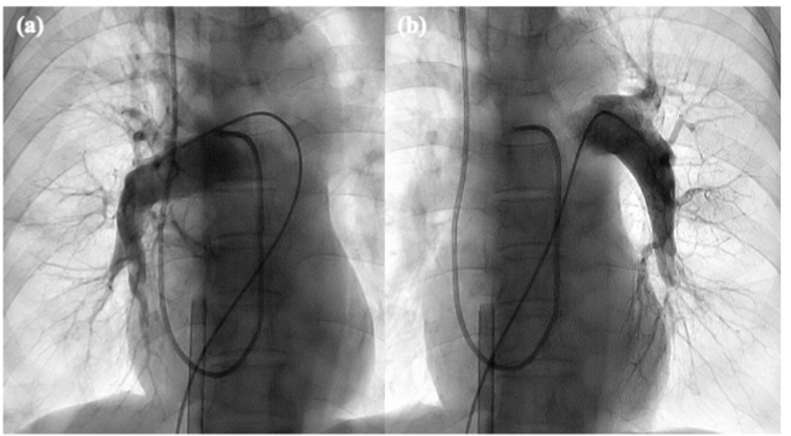
Pulmonary angiography before BPA revealed only a small fresh bloody thrombus in the right A1 and left A9 and chronic thromboembolic lesions of webs, subtotal occlusion, and total occlusion in the bilateral pulmonary arteries at segmental levels.

**Fig. 5:**
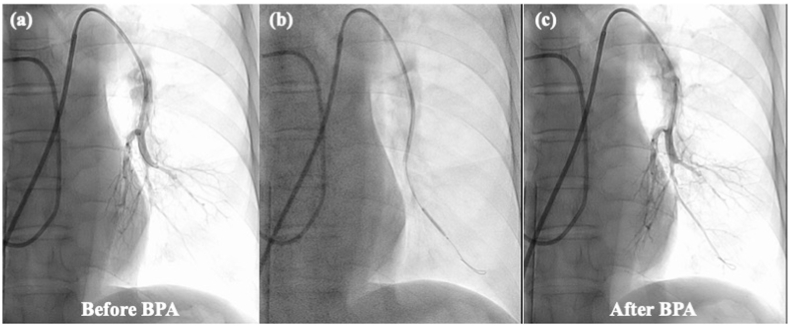
The comparison of pulmonary angiography. (a): pulmonary angiography of left pulmonary artery before BPA; (b): pulmonary angiography of left pulmonary artery during BPA.; (c): pulmonary angiography of left pulmonary artery after BPA. We traversed the lesion with the guide wire shallowly to prevent wire injury. We approached the lesion with an undersized balloon to prevent high perfusion injury or balloon injury and increased the pulmonary flow in each segment slightly.

A 74-year-old woman was admitted to the hospital with progressive exertional dyspnea in the past few weeks. On admission, echocardiography revealed severe PH with a TR-PG of 100 mmHg. Enhanced CT showed a small defect of fresh thrombus in the bilateral pulmonary arteries at the segmental or subsegmental levels and signs of CTEPH including intimal thickening and abrupt narrowing and occlusion in the bilateral pulmonary artery (Fig. 3). Laboratory investigations showed a D-dimer level of 9.1 μg/mL. Although anticoagulation therapy with heparinization was initiated, her condition rapidly deteriorated, leading to severe hypoxemia and hypotension. On the day after admission, ECMO was initiated. She was managed with heparinization and was attempted to be weaned off ECMO. Four days after the initiation of ECMO, mPAP was still 55 mmHg and it was impossible to wean off ECMO without any additional interventions. Therefore, we decided to perform rescue BPA. Pulmonary angiography revealed only a small fresh bloody thrombus in the right A1 and left A9 and chronic thromboembolic lesions of webs, subtotal occlusion, and total occlusion in the bilateral pulmonary arteries at segmental levels (Fig. 4). We performed BPA in the right (A3, A5, A9, A10) and left (A8, A9, A10) pulmonary arteries (Fig. 5). Immediately after BPA, her condition stabilized; however, hemoptysis was observed 1 hour later, requiring frequent tracheal suction. Small-vessel injury was suspected despite the careful wiring approach and ballooning, and bleeding was difficult to manage for 2 days. After hemostasis, her cardiorespiratory status improved and ECMO was successfully discontinued 2 days after rescue BPA. The results of RHC performed 16 days after BPA were as follows: mean PAP 35 mmHg and PVR 7.7 wood units. She was discharged with anticoagulation therapy 1 month after rescue BPA. After an additional four sessions of BPA, her hemodynamics showed further improvement with a mean PAP of 18 mmHg and World Health Organization functional class I.

## 4 Discussion

PE is a life-threatening condition. Treatments can range from anticoagulation therapy alone, systemic thrombolysis, catheter embolectomy, surgical embolectomy, and/or mechanical circulatory support such as ECMO [4]. CTEPH is a fatal disease if left untreated. Although the exact epidemiology of CTEPH is unknown, it has been suggested to involve recurrent embolic episodes after a sufficiently treated PE, in situ propagation of the thrombus into branch pulmonary vessels, and large- and small-vessel vasculopathy caused by incomplete resolution of the initial embolism(3). DiChiacchio et al. reported that more than one-third of patients who underwent acute surgical pulmonary embolectomy also had chronic thromboembolic disease confirmed by intraoperative endarterectomy; however, it is often unrecognized [5]. In our cases, small acute embolism finally deteriorated the hemodynamic states of the CTEPH patients, requiring ECMO. Pulmonary angiography or enhanced CT scan at the time of initiation of ECMO showed many distal CTEPH lesions of the webs or total occlusion; however, only a small fresh thrombus was observed. Even though we started anticoagulation therapy, their condition never improved. Because of these CT images and clinical courses, the main cause of these severe dynamic status was supposed to be distal CTEPH not acute PE. Hence, there was no indication for thrombolysis. Lee et al. reported a case of acute PE on chronic pulmonary thromboembolism requiring ECMO that was treated with pulmonary embolectomy and PEA [6]. PEA should be considered for operable CTEPH. BPA is a promising treatment strategy for the most inoperable CTEPH. Many reports have supported the efficacy and safety of BPA [7,8]. However, severe hemodynamics is known as a risk factor for BPA complications, including severe pulmonary vessel injury [8]. The efficacy and safety of rescue BPA in very severe patients with ECMO support remains to be established. Nakamura et al. suggested that BPA may be a rescue option for distal-type CTEPH patients who do not respond to PEA and require mechanical support [9]. However, Collaud et al. reported three cases of rescue BPA for failure of PEA with ECMO support, and two out of three patients died of multiple organ failure [10]. Indeed, the patient in Case 2 in our study had small pulmonary vessel injury, resulting in severe alveolar bleeding, even with a very careful wiring approach and dilatation with a small balloon. The condition of the pulmonary vessels was presumed to be very fragile, and it was difficult to control bleeding under ECMO support and anticoagulation therapy; even a small-vessel injury might provoke fatal complications. In our cases, we crossed the lesion with the guide wire shallowly to prevent wire injury. When there is a need to advance the wire distally, the knuckle technique was utilized. In addition, we approached the lesion with an undersized balloon (2 mm or 3 mm) to prevent high perfusion injury or balloon injury and tried to increase the pulmonary flow in each segment slightly. As it is less effective in each individual segment, several segments and lobes are targeted in a single session. Thanks to rescue BPA, the patients’ hemodynamic status drastically improved and they could return to their daily lives. The safety of rescue BPA under very severe conditions remains questionable; however, it should be considered as a rescue option in the absence of treatment alternatives.

## Funding

None.

## Disclosures

The authors have reported that they have no relationships relevant to the contents of this paper to disclose.

## Declaration of competing interest

The authors declare that they have no known competing financial interests or personal relationships that could have appeared to influence the work reported in this paper.
